# Porous Graphene Composite Polymer Fibres

**DOI:** 10.3390/polym13010076

**Published:** 2020-12-27

**Authors:** Jubair Ahmed, Tanveer A. Tabish, Shaowei Zhang, Mohan Edirisinghe

**Affiliations:** 1 Department of Mechanical Engineering, University College London, London WC1E 7JE, UK; m.edirisinghe@ucl.ac.uk; 2 College of Engineering, Mathematics and Physical Sciences, University of Exeter, Exeter EX4 4QF, UK; 3 UCL Cancer Institute, University College London, London WC1E 6DD, UK; s.zhang@exeter.ac.uk

**Keywords:** porous graphene, polymer, fibres, polycaprolactone

## Abstract

Since the isolation of graphene, there have been boundless pursuits to exploit the many superior properties that this material possesses; nearing the two-decade mark, progress has been made, but more is yet to be done for it to be truly exploited at a commercial scale. Porous graphene (PG) has recently been explored as a promising membrane material for polymer composite fibres. However, controlling the incorporation of high surface area PG into polymer fibres remain largely unexplored. Additionally, most polymer-graphene composites suffer from low production rates and yields. In this paper, graphene-loaded microfibres, which can be produced at a very high rate and yield have been formed with a carrier polymer, polycaprolactone. For the first time, PG has been incorporated into polymer matrices produced by a high-output manufacturing process and analysed via multiple techniques; scanning electron microscopy (SEM), Raman spectroscopy, Fourier-transform infrared spectroscopy (FTIR) and X-ray diffraction (XRD). Raman spectra showed that single layer graphene structures were achieved, evidence for which was also backed up by the other techniques. Fibres with an average diameter ranging from 3–8 μm were produced with 3–5 wt% PG. Here, we show how PG can be easily processed into polymeric fibres, allowing for widespread use in electrical and ultrafiltration systems

## 1. Introduction

Graphene is a monatomic sheet of carbon atoms which is found tightly packed in a hexagonal crystal lattice [1]. In 2004, adhesive tape was used to isolate a single sheet of graphene that sparked a new wave of interest in graphene and its counterparts, owing to its remarkable properties [2]. Graphene sheets were found to have new and unique properties such as being able to absorb a larger than normal fraction of incident white light (photoacoustic absorption of 2.3%), having very high thermal conductivity between 4840 to 5300 W/mK, electrical conductivity of up to 2000 S/cm and a Young’s modulus of over 1 TPa [3,4,5,6]. Because of these excellent properties relating especially to its optical, thermal, electrical and mechanical features, graphene is potentially a superior replacement medium for current industrial application standards, such as in semiconductors, electromagnetic shielding, healthcare, high-bandwidth wiring systems, fibre optics, material reinforcement and even heat dissipation for advanced hardware cooling [7,8,9,10,11,12]. Graphite is desirable in the single graphene sheet level as the excellent properties become even more pronounced [13].

Porous graphene (PG) has recently been discovered to have refined properties compared to standard graphene due to the mesoporous nature and high specific surface area, which allows for diffusion of ions and molecules [14,15,16]. Highly porous graphene nanosheets are comprised of few-layered graphene sheets which benefit from high specific surface area, superhydrophobicity, optical transparency, good chemical stability and resistance to oxidation [17]. Typically, in the production of PG, high cost and high energy methods such as ion beam bombardment and chemical etching are required [18,19]. A facile and economical production route for PG has been previously reported by us, which utilised lower temperature thermal treatment of reduced graphene oxide; bringing small edge effects into the nano-porous graphene [20,21]. This novel synthesis route forms graphene nanosheets with a specific surface area of over 650 m^2^/g, and due to the more cost-effective attribute of this graphene, it is a much more suitable contender for bulk processing and potential industrial scale up.

Similar to carbon nanotubes, graphene is difficult to bulk process into fully functioning nanocomposite systems [22,23]. Because of its monoatomic nature, graphene-based materials need to be formed at very low thicknesses. Successful incorporation of graphene into fibres could serve to replace current standards, for example silicon in semiconductors [24]. Here, we present a solution-based method for the large-scale production of PG loaded into polycaprolactone (PCL) polymeric fibres. By dispersing PG into a polymer suspension, fibres can be produced at a fast pace and can be suitable for many applications such as filtration and bio-sensing [25,26]. Pressurised gyration, which was invented in 2013, is a hybrid fibre forming technique, which utilises simultaneous centrifugal rotation and infused gas pressure [27,28,29,30]. This technique is capable of producing small-diameter fibres with high-throughput. Pressurised gyration is capable of producing over 1 g of graphene-loaded fibres in under 10 seconds. The solution-based processing of PG serves to form secondary materials with the excellent properties of graphene, whilst having the potential of meeting large industry demands.

In this work, PG-PCL composite fibres were produced via pressurised gyration which allows for the large-scale production of the polymeric composites whilst being able to optimise and tune surface topography and fibre morphology. The PG composite fibres where characterised for their ability to successfully incorporate PG into their polymer matrix. Single layer graphene fibres were desired as that form maximises the apparent properties of the material.

## 2. Materials and Methods

### 2.1. Materials

**Figure 1:**
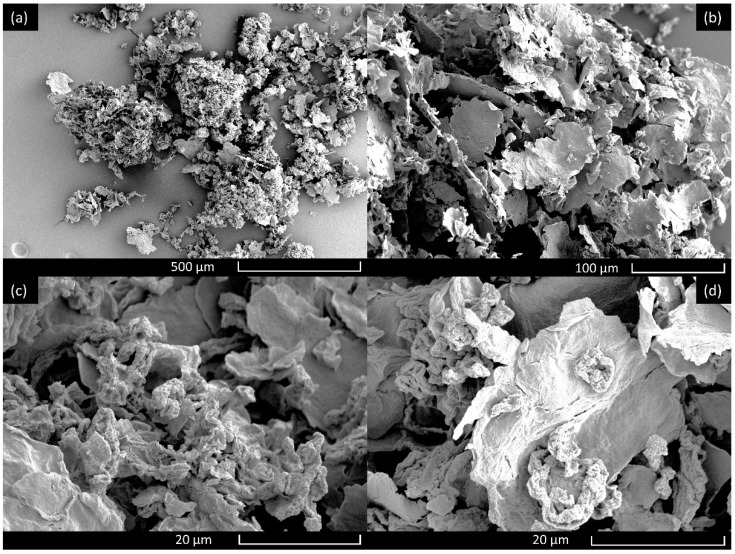
High magnification images of the unprocessed porous graphene shown at (**a**) 85 × magnification, (**b**) 320 × magnification, (**c**) 2100 × magnification and (**d**) 2300 × magnification.

PCL pellets, Mw 80,000 (Sigma-Aldrich, Gillingham, UK) were used as the carrier polymer in order to form graphene-loaded fibres. Chloroform, CAS: 67-66-3, (Sigma-Aldrich, Gillingham, UK) was used as to dissolve the pellets to form a homogenous polymer solution. PG were synthesised by following our previously reported method and can be seen under scanning electron microscope in (Figure 1) [20,21]. Briefly, in the first step, graphene oxide was prepared using modified Hummers method [31]. Exfoliated graphite oxide flakes (of lateral size ~0.5–20 μm and ~1.5 nm thickness) were prepared by oxidising graphite flakes in concentrated H_2_SO_4_, in the presence of NaNO_3_, H_2_O_2_, and KMnO_4_. Graphite oxide was further exfoliated to form graphene oxide (of size 0.8–1 nm). Hydrazine was used as a reducing agent to reduce graphene oxide into reduced graphene oxide (average thickness of 1.5 nm). To obtain PG (average pore size of 3–5 nm), the filtered product of reduced graphene oxide was oven-dried in a vacuum overnight. Thermal treatment was then applied at 200 °C in Argon for a duration of 12 h. The surface morphology (using high-resolution transmission electron microscopy), surface area study (using Brunauer–Emmett–Teller-BET theory method), composition and structural analysis (using X-ray photoelectron spectrometer—XPS, X-ray diffraction, Fourier transform infrared (FT-IR) spectroscopy and Raman spectroscopy) of PG have been shown in detail in our previous study [20].

### 2.2. Preparation of Graphene Polymer Solution

As-prepared PG powder was weighed (3, 4 and 5 wt%) into chloroform where it was suspended, ready for homogenisation. Branson SFX 550 Sonifier (Cole-Parmer, Eaton Socon, UK) was used to disrupt the graphene platelets within chloroform to form a fine suspension of graphene, the output was set to 65% (~325 Watts), and the solution was subjected to ultra-sonication for 6 h. PCL pellets were then added to the PG suspension following ultrasonication to allow thorough dissolution of the polymer. The polymer solutions were stirred mechanically for 24 h at room temperature (20–22 °C).

The prepared solutions were subjected to surface tension measurements using a calibrated tensiometer (Tensiometer K9, Kruss GmbH, Hamburg, Germany). In these measurements, the Du Nouy ring method was used to attain the surface tension, a glass vial was filled with polymer solution and a platinum-iridium ring with a diameter of 60 mm was submerged into each polymer solution. The ring was then slightly raised to enable a fluid meniscus to form, the variation in the forces was measured using a force tensiometer which gave the surface tension values. These readings were carried out at ambient temperature (22–24 °C) and were repeated five times to find the average surface tension values for each solution and concentration.

The viscosity of the solutions was also measured using a Brookfield Viscometer DV-III (Brookfield, Middleboro, MA, USA). A small-sample spindle was used with a polymer volume of 3 mL. The samples were all taken at the same shear rate of ~5 Pa which ensured comparability between the samples. All viscosity measurements were done under ambient conditions (22 ± 2 °C) and repeated three times to give an average value.

### 2.3. Preparation of Graphene-Loaded Fibres

**Figure 2:**
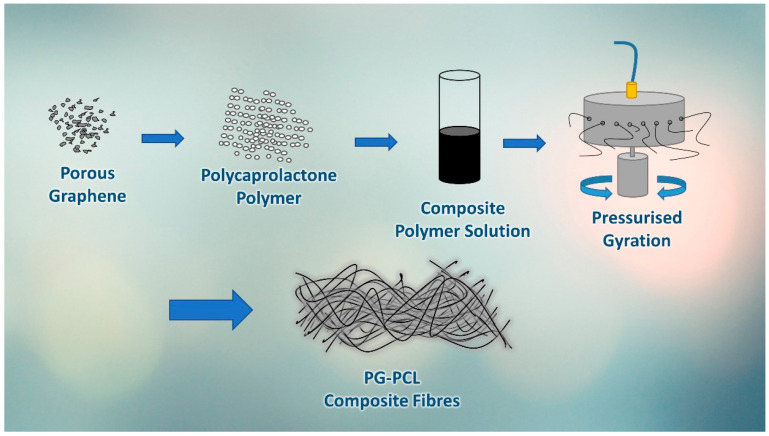
Schematic diagram representing major steps in the production of porous graphene (PG) composite polymer fibres.

Pressurised gyration, a fibre forming technique which combines centrifugal rotation with an inlet gas pressure was used to form the graphene-loaded fibres. A volume of 4 mL of the polymer solutions was placed in the gyration vessel and subjected to a rotation speed of 30,000 rpm at an operating pressure of 0.1 MPa. The production process was carried out at ambient conditions (20–22 °C, 45–55% relative humidity). The essential steps for the production of PG-PCL composite fibres in this paper are summarised in (Figure 2).

### 2.4. Characterisation of Fibres

Following the production of the fibres, they were collected, placed on aluminium studs and gold sputter coated for 180 s (Q150R ES Quorum Technologies Ltd., Laughton, UK). The gold coated samples were then analysed via scanning electron microscopy (SEM) (Hitachi S-3400n, Tokyo, Japan) using an operating voltage of 5 kV. The SEM images were used to detect the structure of the composites; 100 fibres were measured at random, and the mean diameter was calculated using Image J software. The frequency distribution of the fibre diameters was modelled using OriginPro software. Raman spectra of samples were measured in backscattering mode at a 532 nm laser excitation and 6 mW power. Samples were placed on glass slides to collect Raman spectral signatures. FTIR spectra were measured using a spectrometer in the wavenumber range of 4000–500 cm^−1^ (Bruker Optics Tensor-27 IR, Ettlingen, Germany). The samples were prepared by mixing composites with KBr (of spectroscopic grade) to make pellets of about 2 mm in thickness and 5 mm in diameter. X-ray diffraction (XRD) analysis of composites were measured using Cu Kα radiation (at 40 kV and –40 mA).

## 3. Results and Discussion

### 3.1. Characterisation of Fibres

The production of PG-loaded fibres relies heavily on the produced polymer solutions. With higher loading of PG, solution behaviour is expected to deviate as the solution becomes more saturated by the dispersed PG. The PG sheets and the polymer were left to dissolve in tandem, allowing for the polymer chain to fully incorporate with the PG dispersion, for this reason solution properties will have a discernible effect on the fibre morphology.

Solution rheology and other solution characteristics heavily influence the formation of the polymer jet formation in the pressurised gyration spinning process. It is the behaviour of the jet which erupts from the gyration orifices that is responsible for the fibre forming mechanism. The emerging jet dries due to being in contact with the environment, and fibre production is therefore a result of the polymer extrusion the jet undergoes from the orifice to the collector. Without the addition of PG, the average surface tension of the PCL solution was 27.8 mN/m, which is due to using chloroform as the solvent and which is in line with other studies [32,33]. The addition of PG to the polymer solution results in a rise of surface tension from 27.8 to over 35 mN/m, this is likely due to the very high surface energy of carbon-based materials which increases the net inward cohesive force at the surface of the polymer solution [34,35]. There is a slight increase in surface tension of the solutions with higher loading of PG, again likely due to the higher amount of high surface energy materials in the solution.

The concentration of the polymer solution often determines its viscosity, which is typically a proportional increase and therefore rises with higher concentrations [36]. We see that the absence of PG leads to an average viscosity value of 7625 mPa s for the PCL polymer solution, and further addition of PG actually results in the reduction of viscosity. This is a phenomenon which is not commonly seen; the increase of concentration generally leads to an increase in viscosity. One explanation for the reduction in polymer viscosity could be that it is due to the presence of radical degeneration from the PG, which happens when produced radicals react with the polymer backbone, resulting in a reduction of molecular weight [37]. A study into the viscosity of graphene oxide nanoparticles dispersed in water however, did not observe the reduction of viscosity with an increase in graphene concentration [38]. The study concluded that the viscosity was due to particle concentration and temperature, and this suggests that the reduction in viscosity is due to the interaction of the PCL polymer backbone and PG. The unusually low viscosity of graphene has also been theorised to be due to the presence of electronic turbulence. The unique aspect ratio and dimensions of graphene nanoparticles allow it to behave irregularly compared with other nanoparticles [39]. Graphene nanoparticles reduce the intermolecular interaction within the solution, and this could be attributed to the sliding of the nanoparticles which reduce the friction and therefore the measured viscosity. Another study found that by increasing the concentration of reduced graphene oxide, the resulting solution has a 3% reduction of its surface tension followed by a reduction in apparent viscosity [40]. The addition of PG into the polymer solution may also act as a secondary fluid which improves the hydrophobicity of the particles, and this results in an increase in free water in the suspension that significantly reduces the viscosity and yield stress [41]. It is observed that higher loading of PG significantly reduces the viscosity of the polymer solution and at 5 wt%, and the viscosity is less than half that of the original PCL solution.

**Figure 3:**
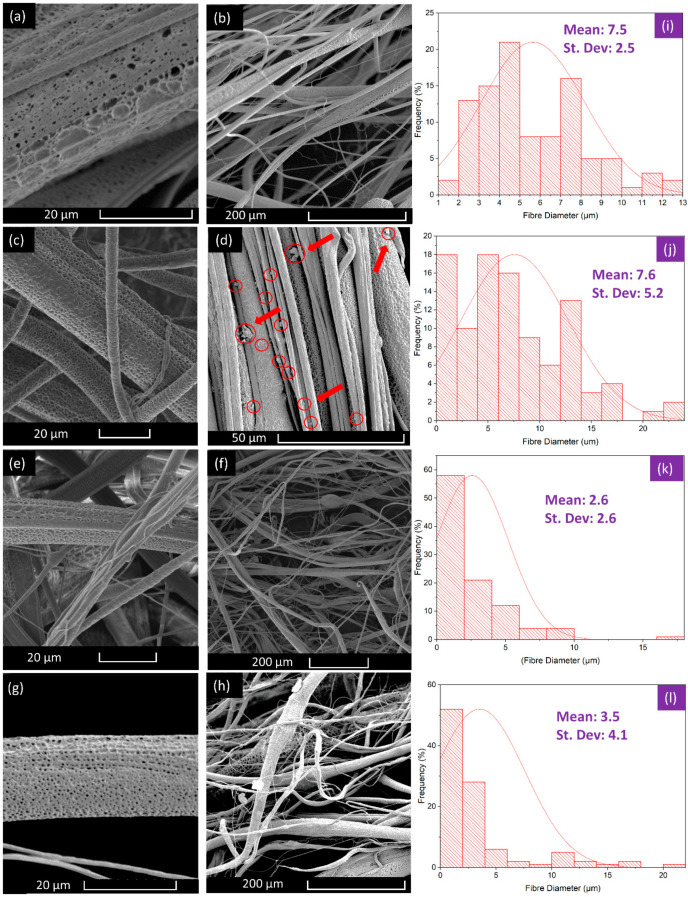
Scanning electron microscopy (SEM) images of fibres; (**a**,**b**) 15% pristine polycaprolactone (PCL) fibres without the incorporation of PG; (**c**,**d**) 3 wt% PG incorporated fibres showing surface porosity and unidirectional alignment; red circles and arrows indicate presence of surface particles; (**e**,**f**) 4 wt% PG incorporated fibres showing surface topography and random orientation alignment and (**g**,**h**) 5 wt% PG incorporated fibres with surface and full view; all images come with accompanying diameter distribution graphs, (**i**–**l**). For each graph 100 fibre strands were measured at random.

High magnification images of the produced fibres allow for the study of fibre diameter distribution, fibre topography and alignment. (Figure 3) shows SEM micrographs of both the full view of the fibre strands and higher magnification images of the fibre surface. From these images, diameter distribution histograms have been deduced which also show the uniformity of the fibres at particular concentrations.

Morphological features of fibres are largely due to the PCL-PG solution properties and the working parameters of the production process. Higher rotational speeds lead to greater extension of the emerging polymer jet which leads to thinner deposited fibres [42]. The fibres presented here were all spun under the same working conditions to ensure that differences in morphology were due to solution characteristics only. Pristine PCL fibres had an average fibre diameter of 7 μm, owing to their high molecular weight and viscosity. Figure 3a,b, revealed that the fibres are mostly aligned unidirectionally which can be a benefit in electrical and optical systems where unidirectional reinforcement increases the front-to-back strength [43,44]. At 3 wt% PG, the fibre diameter remains similar to that of the pristine PCL fibres, and this is caused by the similarity in viscosity values.

As the viscosity of a polymer solution decreases, a downward trend of reduced fibre diameters are observed [45]. The 4 wt% fibres had a significantly lower viscosity and also had a substantially lower fibre diameter. At lower viscosities, there are fewer polymer chain entanglements, and thus, the emerging polymer jet is thinner [46]. However, at 5 wt%, the fibres presented a slight increase in the average diameter, and this could be due to the build-up of additional PG layers on the fibre surface or the inconsistencies afforded by the low solution viscosity and polymer chain entanglement on the forming technique.

All the fibres shown here have been observed to have a porous surface. These pores are the result of using a volatile compound (chloroform) as the solvent. As the chloroform evaporates rapidly, it causes a temperature difference on the fibre surface which leads to condensation of droplets which then evaporate to leave behind nano to micro pores [47]. These pores can greatly increase the surface area to volume ratio making them suitable for applications in hydrogen storage materials for example, where higher surface area will increase the hydrogen absorption capacity [48]. Furthermore, (Figure 3d) shows the 3 wt% fibres with particles on the surface. as indicated by red arrows and circles. These particles correspond with the micrographs of the PG, meaning that some of the graphene has been dispersed onto the surface of the fibre, potentially advantageous as biosensor components and in producing antimicrobial surfaces [49,50,51,52,53,54].

The PG have pores in the size range of 3–5 nm, and these characteristics have been discussed previously [20]. The fibres also contain surface pores due to solvent evaporation as discussed above, and these pores were found to be about 500 nm in diameter. Irrespective of the PG loading shown in (Table 1), in some instances, the pores penetrate deep into the fibre [47]. The surface pores are therefore beneficial in exposing the PG layers to the environment, which is useful in antimicrobial applications [55,56].

### 3.2. Chemical Alaysis of Fibres

**Figure 4:**
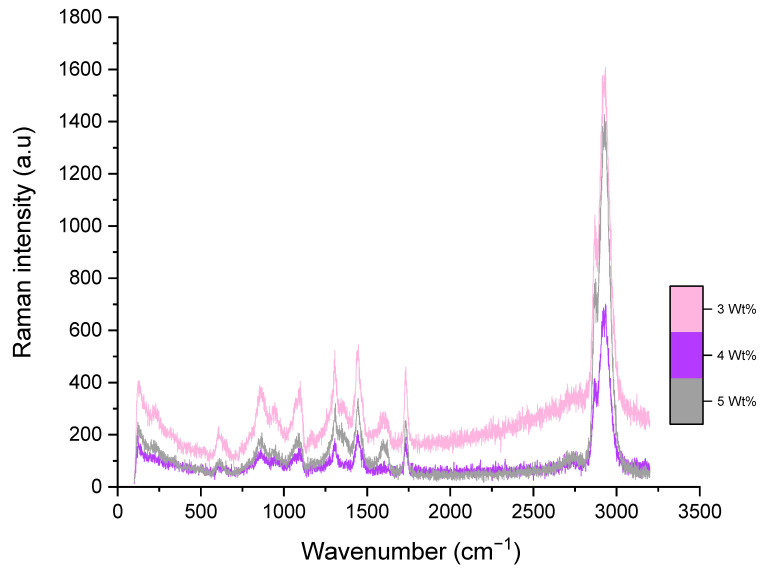
Raman spectra of 3, 4 and 5 wt% PG-PCL composite fibres.

The Raman spectrum of the pristine PCL fibres is displayed in (Figure 4). Raman active bands and their corresponding positions are listed in (Table 2). CH_2_ antisymmetric and symmetric stretching bands are known to be apparent at 2920 and 2868 cm^−1^. The characteristic bands of PCL seen at 1725 cm^−1^ and 1110 cm^−1^ and are designated to νC=O and νCOC, which reveal polymer crystallisation. A weak band at 1725 cm^−1^ and a strong band at 1735 cm^−1^ represent the crystalline and amorphous nature of PCL (as shown in Table 2) [57]. A region of 1287–1306 cm^−1^ is assigned to coupled CH_2_ wagging vibrations. Bands at 1440 (δCH_2_) and 915 (νC–COO) cm^−1^ reveal the crystalline nature of PCL [58]. The decrease in band intensities at 1725 (νC=O; cryst.), 1442 (δCH_2_; cryst.) and 1067 cm^−1^ (νCOC; cryst.) reveal the development of amorphous segments near the surface [58,59]. The band expansion within an amorphous nature and respective reduction in crystallinity represent the disordered morphology of PCL.

Raman spectroscopy showed distinct differences in crystallinity between the pristine PCL and the PG composite fibres. The characteristic graphene G-band peak at 1587 cm^−1^ using a 532 nm excitation laser illustrates that the 3 wt% and 4 wt% PG composite fibres contain a single layer of graphene along their surface [60,61]. As the G peak experiences a shift with additional layers of graphene, it is expected that 5 wt% PG composite fibres contained more than a single layer of graphene, also supported by its larger apparent thickness [62]. Considering the changes expected in sp^2^ hybridisation of PG during the fabrication process of composite fibres with pressurised gyration, Raman spectroscopy was used to study the defective structures of composite fibres. Raman spectra revealed that there is a shift in the vibrational bands of graphene, clearly showing that the graphene layers are in contact with the polymer. Furthermore, the considerable shift in the 2D Raman peak demonstrates that the composites were robust and that no interfacial issues were encountered.

**Figure 5:**
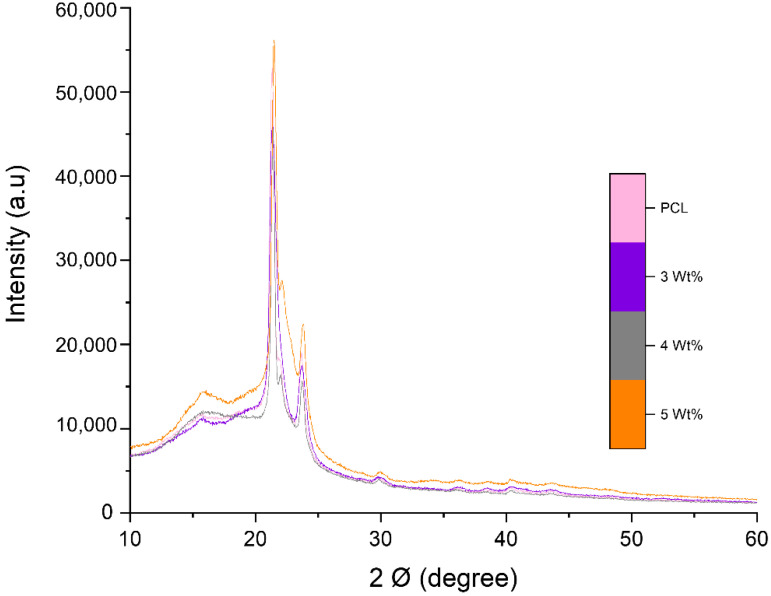
XRD patterns of pristine PCL fibres along with 3, 4 and 5 wt% PG-PCL fibres.

XRD peaks of different concentrations of PG within the fibres revealed that the (002) peak of PG-loaded fibres was extended and had an evidently decreased mode, showing that composites were comprised of single-layered graphene [63] (Figure 5). The diffraction patterns of the composites show characteristic peaks of graphene and demonstrates that PG could play a role as an agent for polymer crystallization.

**Figure 6:**
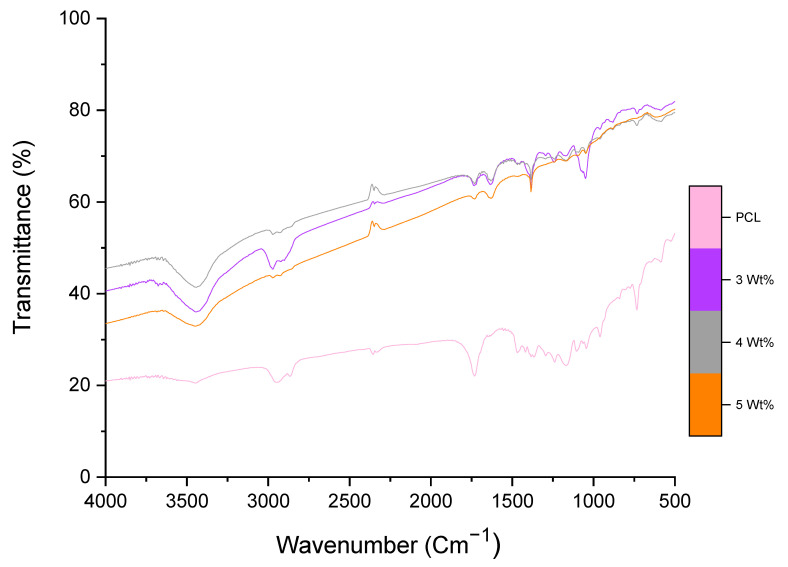
Fourier Transform Infrared (FTIR) spectra comparing the chemical compositions of pristine PCL fibres along with 3, 4 and 5 wt% PG-PCL fibres.

The FTIR spectrum of PG has been reported previously [20]. The distinct peaks of PG were at 1735 and 1072 cm^−1^ conforming to the C=O and C–O–C stretching vibrations from carbon and epoxy functional groups, respectively [31,64]. Figure 6 demonstrates the FTIR spectra of PCL and PG-loaded PCL. PCL showed a peak near 3500 cm^−1^ and a strong band at 1745 cm^−1^, corresponding to hydroxyl and ester functional groups, respectively. The other distinct peaks of PCL were asymmetric and symmetric CH_2_ bonds corresponding to bands at 2940 and 2860 cm^−1^, respectively [65]. The FTIR spectra of PG-loaded PCL showed a significant sharpness at 3500 cm^−1^ referring to OH groups coming from PG molecules and reduction in the intensity at 1700 cm^−1^, this confirmed the increase of PG molecule covalent coupling onto the surface of PCL [66]. FTIR has been used to analyse the interconnected features of PG-polymer composite fibres and to study the cross-linking of functional groups such as OH, CO, COC and other epoxy groups from PG edges to fibres. PG-fibres demonstrate the reordering of bonding in their networks. FTIR analysis of fibres further demonstrates the robustness of fibres and also the cross-linking of PG to fibres at correct ratios.

The combination of the analysis techniques showed that PG was successfully incorporated into the PCL solution and was able to form fibres. These assemblies, as seen from SEM images, were correctly identified as fibres which differed in fibre diameter distribution ranges but ultimately all had surface pores. Raman studies and XRD analysis eluded to single-layer formation of graphene being present on the fibres. FTIR spectroscopy confirmed the presence of graphene on the composite samples and established that higher loading of PG in the polymer solutions leads to higher and more successful incorporation of graphene into the fibres. These graphene-based composite fibres could be utilised to release and store pharmaceuticals, genetic material and biological molecules and can be used in biosensing, the treatment of many diseases, as well as used in wound healing and to treat viral infections.

## 4. Conclusions

It was shown that the addition of PG loading causes the polymer solution to reduce in viscosity, and this in turn leads to smaller fibre diameters being achieved as the PG content increased from 3 to 5 wt%. SEM images showed the fibres to have surface porosity, which increases the surface area to volume ratio of the fibres. All fibre samples from as low as 3 wt% showed proper loading of PG. It is shown that the chemical properties of the PG-composite fibres do not differ considerably from 3 to 5 wt%. However, at higher concentrations, the fibres have a smaller diameter, providing a larger surface area to volume ratio. The surface tension of the polymer solutions did not significantly change with the increase in PG concentration. Raman spectroscopy unveiled that the 3 and 4 wt% PG-PCL fibres were encased with single layer graphene whilst higher concentrations begin to show multiple layers. By comparing the XRD analysis of the different samples, the 002 peak indicated that the PG-PCL composite fibres where composed of single layer graphene, further corroborating the idea that these fibres contained only single sheets. FTIR spectroscopy confirmed the presence of graphene within the PG composite fibres and also demonstrated that higher loadings of PG successfully lead to higher amount of graphene being taken up by the fibres. The first ever successful production of PG-composite polymeric fibres was demonstrated here with a production method that is capable of producing kilogram quantities of fibres in an hour. This work shows great promise in the manufacture of graphene composite materials with single layer fibre coatings which can especially be exploited in electrical systems due to the high conductivity it affords.

## Figures and Tables

**Table 1:** Solution characteristics for the studied polymer solutions all made with 15% (*w/v*) polycaprolactone.

PG Concentration (wt%)	Surface Tension (mN/m)	Viscosity (mPa·s)
0	27.8 ± 0.6	7625 ± 86
3	35.8 ± 1.0	7366 ± 78
4	36.8 ± 1.2	3679 ± 155
5	37.0 ± 1.1	3039 ± 160

**Table 2:** Collected Raman bands (cm^−1^) and their corresponding assignments for PCL-based graphene nanocomposite at 532 nm laser excitation [57,58,59].

PCL-Porous Graphene-Based Composite Raman Bands [cm^−1^]			Band Assignment
PCL-PG 3%	PCL-PG 4%	PCL-PG 5%	
869	877	872	ν(C–COO); amorph
956	957	950	ν(C–COO);
1099	1097	1090	ν(COC); amorph
1198	1287	1285	ω(CH_2_); cryst
1306	1305	1309	ω(CH_2_); cryst & amorph
1350	1356	1348	δ(CH_2_)
1440	1446	1448	δ(CH_2_); cryst
1576	1576	1616	δ(CH_2_)
1728	1729	1731	ν(C=O); cryst
1733	1730	1736	ν(C=O); amorph
2893	2893	2875	ν(CH_2_)
2930	2930	2929	C–H stretching ν(CH_2_)

## Data Availability

Data available in a publicly accessible repository.
